# A Denoising Method for Mining Cable PD Signal Based on Genetic Algorithm Optimization of VMD and Wavelet Threshold

**DOI:** 10.3390/s22239386

**Published:** 2022-12-01

**Authors:** Yanwen Wang, Peng Chen, Yongmei Zhao, Yanying Sun

**Affiliations:** 1 School of Mechanical, Electronic & Information Engineering, China University of Mining and Technology-Beijing, Beijing 100083, China; 2 CHN Energy Technology & Economics Research Institute Co., Ltd., Beijing 100083, China

**Keywords:** PD denoising, VMD, wavelet threshold, genetic algorithm, mining cables

## Abstract

When the pulse current method is used for partial discharge (PD) monitoring of mining cables, the detected PD signals are seriously disturbed by the field noise, which are easily submerged in the noise and cannot be extracted. In order to realize the effective separation of the PD signal and the interference signal of the mining cable and improve the signal-to-noise ratio of the PD signal, a denoising method for the PD signal of the mining cable based on genetic algorithm optimization of variational mode decomposition (VMD) and wavelet threshold is proposed in this paper. Firstly, the genetic algorithm is used to optimize the VMD, and the optimal value of the number of modal components *K* and the quadratic penalty factor *α* is determined; secondly, the PD signal is decomposed by the VMD algorithm to obtain *K* intrinsic mode functions (IMF). Then, wavelet threshold denoising is applied to each IMF, and the denoised IMFs are reconstructed. Finally, the feasibility of the denoising method proposed in this paper is verified by simulation and experiment.

## 1. Introduction

The online monitoring of partial discharge (PD) is considered an effective method for checking cable insulation defects and identifying potential faults. It has been widely used in the condition monitoring of power cables [1,2,3]. At present, the ultra-high frequency (UHF) method [4] and pulse current method [5] are commonly used for PD measurement. The center frequency of UHF is 500 MHz. According to different measurement methods, its bandwidth is more than ten MHz or even several GHz. The measurement frequency of the pulse current method is relatively low, usually a few kHz to a few hundred kHz. However, the UHF signal attenuation is serious or even cannot be measured during the transmission of the PD signal in the cable, so the effect of the UHF method in the PD monitoring of cables is not ideal. We believe that the pulse current method is more suitable for the PD monitoring of cables. The pulse current method is mainly subject to noise interference, and it is difficult to separate from the interference signal. To ensure that the PD signal is not distorted as much as possible and to improve the signal-to-noise ratio (SNR) of the PD signal, it is necessary to conduct more in-depth research on the denoising algorithm of the cable PD signal. 

The PD signal has the characteristics of nonlinear, time series non-equilibrium, and wide frequency band distribution, so it is not easy to effectively denoise the PD signal by selecting its frequency band. Currently, the empirical mode decomposition (EMD) method and wavelet threshold method are the main denoising methods for PD [6,7,8]. The EMD method recursively detects the local maximum and minimum values in the signal, which is highly dependent on the extreme point search method. According to the energy rule, the intrinsic mode function (IMF) with a small order is directly discarded, resulting in the loss of some valuable signals. The wavelet threshold method can realize the signal localization in both time and frequency domains simultaneously, which has good time-frequency analysis capability. However, the transient process of local projection will be lost due to the wavelet transform.

In order to solve the shortcomings of EMD and the wavelet threshold method in PD signal denoising, many scholars have made many improvements to these two methods. A partial discharge-based novel adaptive ensemble empirical mode decomposition (Novel Adaptive EEMD, NAEEMD) method is proposed by Tao Jin [9] for noise reduction. After using EEMD to decompose the PD signal, the method adaptively selects the intrinsic mode function for noise reduction reconstruction. Jeffery C. Chan [10] proposes a self-adaptive technique for partial discharge (PD) signal denoising with automatic threshold determination based on EEMD and mathematical morphology. On the basis of mathematical morphology, an automatic morphological thresholding (AMT) technique is developed to form upper and lower thresholds for automatically eliminating the residual noise while maintaining the PD signals. Ramy Hussein [11] proposes a wavelet-based denoising method with a new histogram-based threshold function and selection rule. The proposed threshold estimation technique obtains two different threshold values for each wavelet sub-band and uses a prodigious thresholding function that conserves the original signal energy. Jun Zhong [12] proposes a method without human intervention in choosing the threshold parameters or the decomposition layer numbers.

In 2014, Konstantin Dragomiretskiy proposed Variational Mode Decomposition (VMD) based on EMD [13]. Compared with EMD, VMD has fewer decomposition layers and rigorous mathematical theory, which improves its robustness against noise interference [14,15,16]. The VMD method can retain the transient PD process relatively completely, but its ability to suppress noise is weak. In conclusion, for the PD signal, which is more seriously affected by the field interference signal, the filtering effects of the above methods are less satisfactory when used alone.

After analyzing the advantages of VMD and wavelet threshold, a combined denoising method based on VMD and wavelet threshold is proposed. This method combines the advantages of VMD’s ability to adaptively adjust the center frequency of each mode and the excellent time-frequency analysis ability of wavelet threshold method while avoiding the disadvantages of VMD’s weak noise suppression ability and the transient process of wavelet threshold loss. At the same time, we use genetic algorithm (GA) to optimize the number of modal components and quadratic penalty factor in VMD and determine the two input parameters that can achieve the optimal decomposition of VMD. The structure of this paper is as follows: Section 2 introduces the basic principles of VMD, GA and wavelet threshold method; Section 3 describes the specific process of the proposed denoising algorithm and the process of optimizing the parameters by GA; Section 4 shows the simulation verification results and the comparison of the denoising effect between the proposed method and several different methods. In Section 5, the experimental signal denoising results are described in detail. The conclusion of this paper is given in Section 6.

## 2. Methods and Principles

### 2.1. VMD Method

VMD can adaptively and non-recursively decompose the input signal into multiple IMFs with specific sparse properties. Each IMF has a corresponding center frequency. During the decomposition process, each mode is continuously evaluated to optimize the distribution of each IMF and its center frequency.

The VMD algorithm is actually a solution process for a variational problem. It decomposes the original signal into *K* IMFs *u_k_*(*t*), so that the sum of the estimated bandwidths of each IMF is minimized, and then, the corresponding constrained variation model can be expressed as:

(1) $$\begin{array}{l} \underset{\left\{u_{k}\},\{\omega_{k}\right\}}{\min}\left\{{\sum_{k=1}^{K}\left\|\partial_{t}[[(\delta (t)+\frac{j}{\pi t})*u_{k}(t)]e^{-j\omega_{k}t}]\right\|}_{2}^{2}\right\}\\ s.t.\sum_{k=1}^{K}u_{k}=f \end{array}$$

where {*u_k_*} represents the set of all IMFs, {*ω_k_*} represents the set of center frequencies corresponding to the IMF, $\left(\delta (t)+\frac{j}{\pi t})*u_{k}(t\right)$ is the unilateral frequency spectrum of each eigenmode, obtained by computing its analytic signal through the Hilbert transform, and *f* is the original signal.

To transform the above constrained variational problem into an unconstrained variational problem, the quadratic penalty factor *α* and the Lagrangian multiplier *λ*(*t*) are introduced, and the extended Lagrangian expression is:

(2) $$L(\{u_{k}\},\{\omega_{k}\},\lambda ):=\alpha {\sum_{k=1}^{K}\left\|\partial_{t}[[(\delta (t)+\frac{j}{\pi t})*u_{k}(t)]e^{-j\omega_{k}t}]\right\|}_{2}^{2}+{\left\|f(t)-\sum_{k=1}^{K}u_{k}(t)\right\|}_{2}^{2}+\langle \lambda (t),f(t)-\sum_{k=1}^{K}u_{k}(t)\rangle$$

The alternate direction method of multipliers (ADMM) is used to solve the variational problem, and the optimal solution of the above function is obtained by iteratively updating *u_k_^n^*^+1^, *ω_k_^n^*^+1^ and *λ^n^*^+1^. The value problem of *u_k_^n^*^+1^ can be expressed as:

(3) $$u_{k}^{n+1}=\underset{u_{k}\in X}{\arg\min}\{\alpha {\left\|\partial_{t}[[(\delta (t)+\frac{j}{\pi t})*u_{k}(t)]e^{-j\omega_{k}t}]\right\|}_{2}^{2}+{\left\|f(t)-\sum_{i=1,i\neq k}^{K}u_{i}(t)+\frac{\lambda (t)}{2}\right\|}_{2}^{2}\}$$

where *ω_k_* and *u_i_*_≠_*_k_* represent the latest available update value. Using the Parseval Fourier equidistant transform, the above equation can be transformed as:

(4) $${\overset{\wedge }{u}}_{k}^{n+1}(\omega )=\frac{\overset{\wedge }{f}(\omega )-\sum_{i=1,i\neq k}^{K}{\overset{\wedge }{u}}_{i}(\omega )+\frac{\overset{\wedge }{\lambda }(\omega )}{2}}{1+2\alpha {\left(\omega -\omega_{k}\right)}^{2}}$$

According to the same solution process as *u_k_*, the solution of the quadratic optimization problem of the center frequency is shown in Formula (5):

(5) $$\omega_{k}^{n+1}=\frac{\int_{0}^{\infty }\omega {\left|{\overset{\wedge }{u}}_{k}(\omega )\right|}^{2}d\omega }{\int_{0}^{\infty }{\left|{\overset{\wedge }{u}}_{k}(\omega )\right|}^{2}d\omega }$$

where ${\overset{\wedge }{u}}_{k}^{n+1}(\omega )$ is equivalent to the Wiener filtering result of the current residual $\overset{\wedge }{f}(\omega )-\sum_{i=1,i\neq k}^{K}{\overset{\wedge }{u}}_{i}(\omega )$; and $\omega_{k}^{n+1}$ is the center of gravity of the power spectrum of the modal function.

The VMD algorithm is continuously updated in the frequency domain, and then, the inverse Fourier transform is performed to obtain the time domain result. The specific process of the VMD algorithm can be described as follows:

Step 1: Given the number of modal decompositions *K* and the penalty factor *α*, initialize $\left\{\overset{\wedge }{u_{k}^{1}}\right\}$, $\left\{\overset{\wedge }{\omega_{k}^{1}}\right\}$, $\left\{\overset{\wedge }{\lambda^{1}}\right\}$ and *n*;

Step 2: Update *u_k_* and *ω_k_* in the frequency domain according to Formulas (4) and (5);

Step 3: Update *λ*, and its update formula is as follows:

(6) $$\overset{\wedge }{\lambda^{n+1}}(\omega )=\overset{\wedge }{\lambda^{n}}(\omega )+\tau (\overset{\wedge }{f}(\omega )-\sum_{K}\overset{\wedge }{u_{k}^{n+1}}(\omega ))$$

Step 4: Given the discrimination accuracy *ε*, when it is satisfied $\frac{\sum_{k}{\left\|{\overset{\wedge }{u}}_{k}^{n+1}-{\overset{\wedge }{u}}_{k}^{n}\right\|}_{2}^{2}}{{\left\|u_{k}^{n}\right\|}_{2}^{2}}<\varepsilon$, stop the iteration and output the IMF {*u_k_*}.

### 2.2. Genetic Algorithm

**Figure 1:**
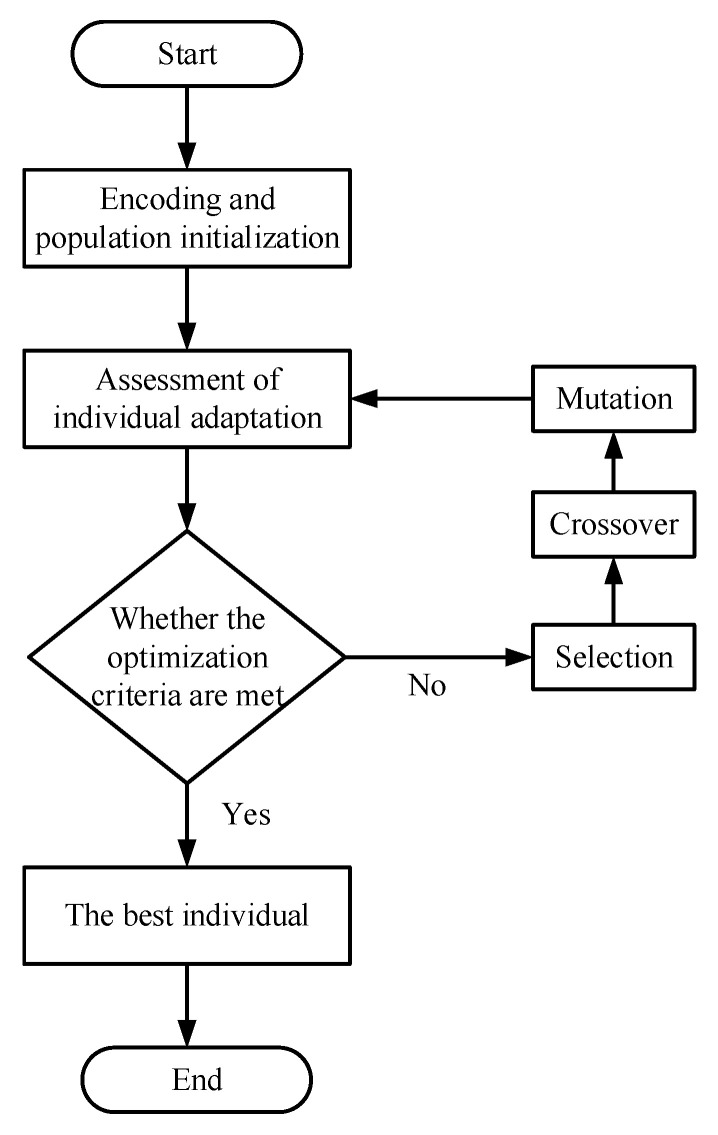
Genetic algorithm flow.

The genetic algorithm is an optimization algorithm that simulates the natural selection and genetic evolution of organisms [17,18,19]. It usually includes three genetic operators: selection, crossover and mutation. The genetic algorithm is an iterative process; each cycle is a generation. In the operation, the inheritance is terminated after a specified number of generations, and then, the optimal chromosome is found among all generations. The genetic algorithm optimization is performed again if the optimal solution is not found. When using the genetic algorithm to solve the optimization problem, it mainly needs to go through six steps: encoding, initial population generation, fitness value evaluation, selection, crossover, and mutation, so that the population evolves into a new generation of a better adaptive population. The specific process of the genetic algorithm is shown in Figure 1.

### 2.3. Wavelet Threshold Denoising

The basic idea of the wavelet threshold denoising method is that a noisy signal can be expressed as the superposition of the original signal and the noise obeying the Gaussian distribution [20,21]. Since the wavelet transform is linear, the wavelet coefficients of the original signal and the noise can be obtained, respectively, after the noisy signal undergoes discrete wavelet transform. Based on the fact that the useful signal and the noise have different statistical properties after the wavelet transform, the original signal’s wavelet coefficients are larger and more significant than the noise’s. Therefore, an appropriate threshold *λ* is found as a criterion for judging whether the decomposed signal is discarded or not. When the decomposition coefficient is less than the threshold *λ*, it is considered that the decomposition coefficient is mainly caused by noise, and the corresponding decomposition signal should be discarded; when the decomposition coefficient is greater than the threshold *λ*, it is considered that the decomposition coefficient is mainly caused by the signal, and the corresponding decomposition signal is processed. Then, wavelet reconstruction is performed to obtain the denoised signal. The specific process of wavelet threshold denoising is as follows:

Step 1: Select the appropriate wavelet basis function and decomposition level and perform wavelet decomposition on the noisy signal.

Step 2: Select an appropriate threshold to properly process the wavelet coefficients. When the decomposed wavelet coefficients are smaller than the selected threshold, it is considered that the wavelet coefficients are mainly caused by noise and should be set to zero. When the wavelet coefficients are greater than the selected threshold, it is believed that the wavelet coefficients are mainly due to the signal.

Step 3: Perform inverse wavelet transform on the processed wavelet coefficients to obtain a denoising result.

Wavelet transform is a new transform analysis method, which transforms the function *f*(*t*) under the wavelet basis, and its expression is:

(7) $$WT_{f}(a,\tau )=[f(t),\psi_{a,\tau }(t)]=\frac{1}{\sqrt{a}}\int_{R}f(t)\psi^{*}(\frac{t-\tau }{a})dt$$

where *ψ*(*t*) is the wavelet basis function; *a* is the expansion and contraction amount; and *τ* is the translation amount. It can be seen from the above formula that the wavelet transform is actually the integral transform of the function, *WT_f_*(*a*,*τ*) represents the wavelet coefficient after the wavelet, and the expression of the inverse transform can be expressed as:

(8) $$f(t)=\frac{1}{c_{\phi }}\int_{0}^{+\infty }\frac{da}{a^{2}}\int_{-\infty }^{+\infty }WT_{f}(u,\tau )\frac{1}{\sqrt{a}}\psi (\frac{t-\tau }{a})dt$$

When thresholding the wavelet coefficients, there are usually two methods: hard thresholding and soft thresholding. Hard thresholding is to keep larger coefficients and zero out smaller coefficients, as shown in Formula (9):

(9) $${\overset{\wedge }{W}}_{j,k}=\left\{\begin{array}{cc} W_{j,k} & \left|W_{j,k}\right|\geq Thr\\ 0 & \left|W_{j,k}\right|<Thr \end{array}\right.$$

Soft thresholding is to set the smaller wavelet coefficients to zero and the larger coefficients to shrink toward zero, as shown in Formula (10):

(10) $${\overset{\wedge }{W}}_{j,k}=\left\{\begin{array}{cc} \mathrm{sgn}(W_{j,k})*(\left|W_{j,k}\right|-Thr) & \left|W_{j,k}\right|\geq Thr\\ 0 & \left|W_{j,k}\right|<Thr \end{array}\right.$$

where *W_j,k_* represents the wavelet coefficient; *Thr* represents the threshold.

## 3. PD Signal Denoising Based on Genetic Algorithm Optimization of VMD and Wavelet Threshold

### 3.1. PD Signal Denoising Process

**Figure 2:**
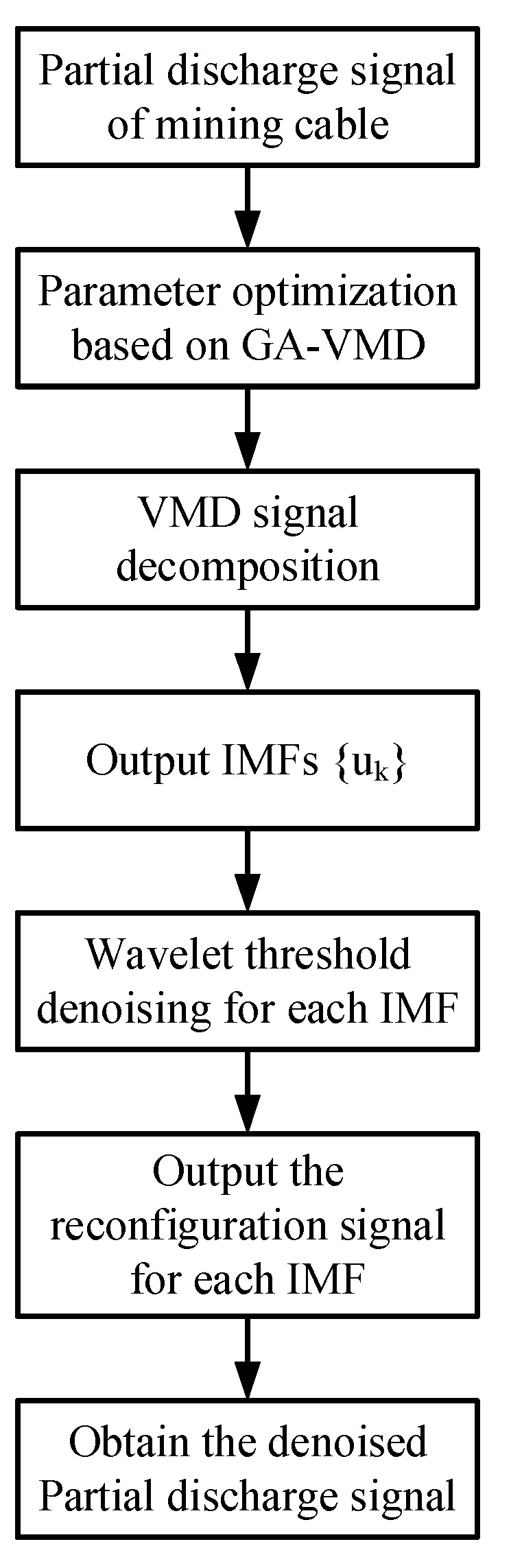
Denoising flow chart of PD signal.

The PD signal has a wide frequency band, and the main frequency is not apparent. It can be seen from the above basic principles that the VMD method can adaptively decompose the PD signal into multiple eigenmodes with center frequencies. It can not only extract the PD signal from the interference but also preserve the transient process of the PD signal as much as possible. According to the basic principles of VMD, genetic algorithm and wavelet threshold, we propose a PD denoising method based on the genetic algorithm optimization of VMD and wavelet threshold. Firstly, the two input parameters of VMD are optimized by genetic algorithm, and the optimal parameter value that can make VMD achieve the best decomposition effect are obtained. Then, *K* IMFs are decomposed by VMD; that is, {*u*_1_, *u*_2_, …, *u_k_*}, wavelet threshold denoising is applied to each IMF to obtain the denoised components of each IMF. Finally, we perform signal reconstruction on the denoised components of all IMFs. The specific denoising process is shown in Figure 2.

### 3.2. Optimization of VMD Parameters Based on Genetic Algorithm

From the theory of VMD, it is known that VMD needs to pre-set the number of modal components *K* when decomposing the signal. The VMD decomposition results obviously differ with different settings of the number of modal components. It has been found that the quadratic penalty factor *α* in the VMD method also has a large impact on the VMD decomposition results. However, the number of modal components *K* and the quadratic penalty factor *α* need to be manually set in advance, and the randomness and uncertainty of the artificial setting will inevitably affect the correctness of the VMD decomposition result. How to choose the appropriate two input parameters is the premise and key to accurately decompose the signal by VMD.

Since genetic algorithm is a direct search optimization method generated by evolution theory and genetic mechanism, it has good global probability search ability. Therefore, this paper uses genetic algorithm to optimize the two input parameters *K* and *α* of VMD and obtain the optimal value. Input parameters. When the genetic algorithm searches for the input parameters of the VMD method, an adaptation function needs to be defined in step 3. The information entropy can well evaluate the sparse characteristic of the signal. The size of the information entropy reflects the uncertainty of the signal. The larger the entropy value, the greater the uncertainty of the signal. The entropy value of *e_j_* (*e_j_* is the signal sequence after demodulation and decomposition) is the envelope entropy, which can reflect the sparse characteristic of the original signal. The envelope entropy *E_e_* of the zero mean signal *x*(*j*) (*j* = 1, 2, …, *N*) can be expressed as:

(11) $$\left\{\begin{array}{c} E_{e}=-\sum_{j=1}^{N}e_{j}\mathrm{lg}e_{j}\\ e_{j}=a(j)/\sum_{j=1}^{N}a(j) \end{array}\right.$$

where *e_j_* is the normalized form of *a*(*j*); and *a*(*j*) is the envelope signal of signal *x*(*j*) after Hilbert transform.

In order to search for the global optimal *u_k_* component combination, we take the local minimum envelope entropy value as the fitness value in the whole parameter optimization process, and we take the minimization of the local minimum envelope entropy value as the final parameter optimization goal.

## 4. Simulation Verification and Analysis

### 4.1. Simulation Results

**Figure 3:**
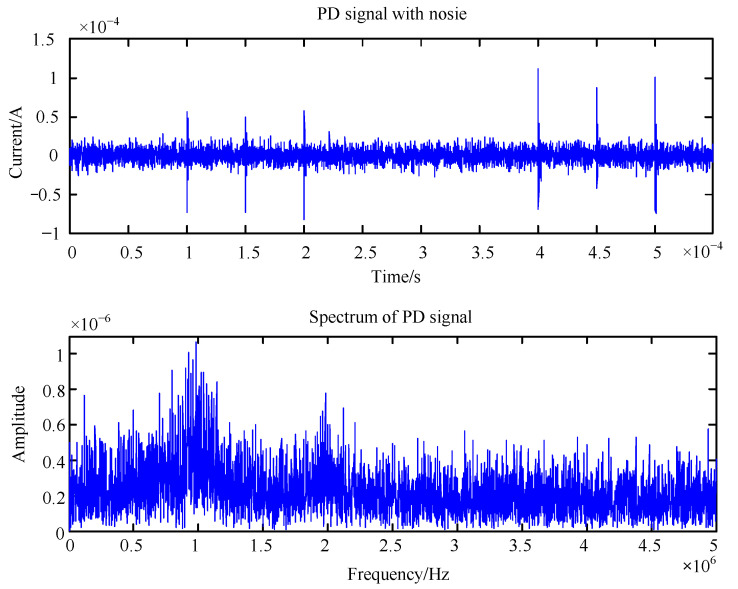
The waveform of the PD signal with noise and its spectrum.

In the PD monitoring site, various random noise disturbances will be generated in electrical systems such as analog circuits, photoelectric conversion, analog/digital conversion, and communication lines, which are expressed in the form of Gaussian white noise signals. The characteristic of white Gaussian noise is that its amplitude is Gaussian distribution N (0, 1), and its power spectral density is uniformly distributed. In order to simulate the pulse signal obtained in the field, the original PD pulse signal is superimposed with Gaussian white noise to obtain a noisy signal. The one-dimensional signal model with noise can be expressed as: *d_i_ = f_i_ + εz_i_, i* = 1, 2, …, *N*. Among them, *d_i_* is the noisy signal, *f_i_* is the “pure” PD pulse signal, *z_i_* is Gaussian white noise, *ε* is the noise level, and N is the signal length. The PD signal is weak, the signals measured on-site has many noise components, and the SNR of the signal is very low. Taking a noisy signal with a signal-to-noise ratio of −2.67 dB as an example, its waveform and spectrum are shown in Figure 3. It can be seen that the PD signal is submerged in noise, which has a wide frequency range and is randomly distributed in the frequency band of the PD signal. The spectral characteristics of the PD signal in the noisy signal are not prominent.

**Figure 4:**
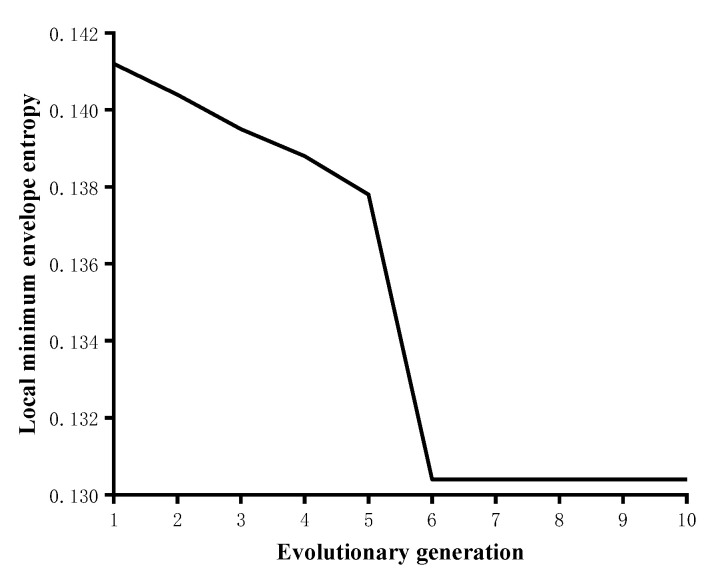
Local minimal envelope entropy values of the simulated signal at different genetic generations.

Figure 4 shows the results of the genetic algorithm for the optimal search of VMD input parameters, which reflects the plot of the local minimal envelope entropy values of the simulated signals at different genetic generations. The minimum value of the local minimal envelope entropy value 0.1307 appears in the 6th generation, and the optimal input parameter (*K*, *α*) = (5, 847) is obtained by the search. Therefore, the number of modal components *K* in the VMD method is set to 5, and the quadratic penalty factor *α* is 847 to decompose the simulated signal.

**Figure 5:**
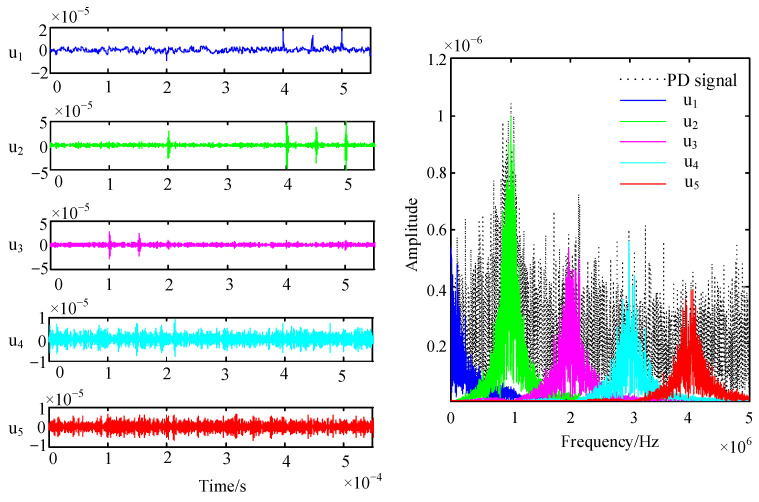
The decomposed eigenmodal components and their corresponding spectra, when *K* = 5.

Figure 5 shows the decomposed eigenmodal components and their corresponding spectra when *K* is taken as 5. It can be seen that the peak of the spectrum of mode *u*_2_ coincides with the peak of the spectrum of the noise-containing signal, and the peak of the spectrum of mode *u*_3_ coincides with the peak of the noise-containing signal near 2 MHz. This shows that the VMD decomposition of the noise-bearing signal is the best at this time.

**Figure 6:**
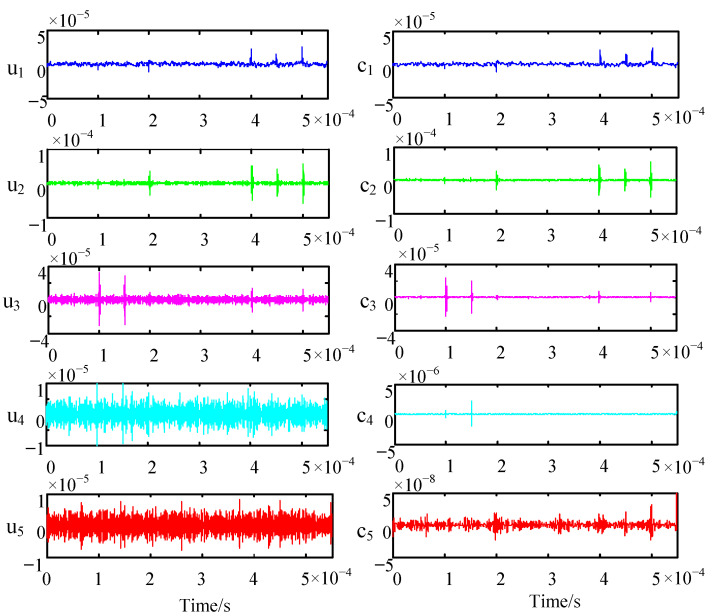
Intrinsic mode functions and wavelet threshold denoising signals.

After determining the values of *K* and *α*, we perform VMD on the noisy signal. Each modal component *u_k_* obtained after decomposition still contains obvious noise interference, so it is necessary to perform wavelet threshold denoising on each mode component *u_k_* separately to achieve better denoising effect. After our careful analysis, the db.4 wavelet is selected as the wavelet base in this paper, the decomposition scale is three layers, and the threshold value is calculated using the fixed threshold estimation method. In order to ensure the smoothness of the signal, a soft threshold function is selected for processing. As shown in Figure 6, the waveforms of each eigenmode *u_k_* are shown on the left, and the corresponding waveforms *c_k_* obtained after wavelet thresholding for each eigenmode are shown on the right.

It can be seen from Figure 6 that noise is significantly suppressed in the reconstructed signal ck obtained by the wavelet decomposition of each eigenmode component *u_k_*. In particular, the PD components in *u*_4_ and *u*_5_ are completely submerged in the noise, and after the wavelet threshold decomposition, the PD components in *c*_4_ and *c*_5_ are obvious. VMD is characterized by the ability to decompose a broadband signal into a signal consisting of multiple narrowbands. Therefore, some scholars have achieved the separation of low-frequency mixed signals or low-frequency noise-laden signals by the selective rounding of IMFs through VMD. However, the characteristic of the PD signal is that its frequency band is extremely wide and it is difficult to find a fixed dominant frequency, so the abandonment of IMFs will lead to the loss of useful signal information. Through the secondary processing of wavelet threshold denoising, the noise interference is effectively removed, and the useful signals in each IMFs are preserved to a great extent.

### 4.2. Comparison of Different Methods

**Figure 7:**
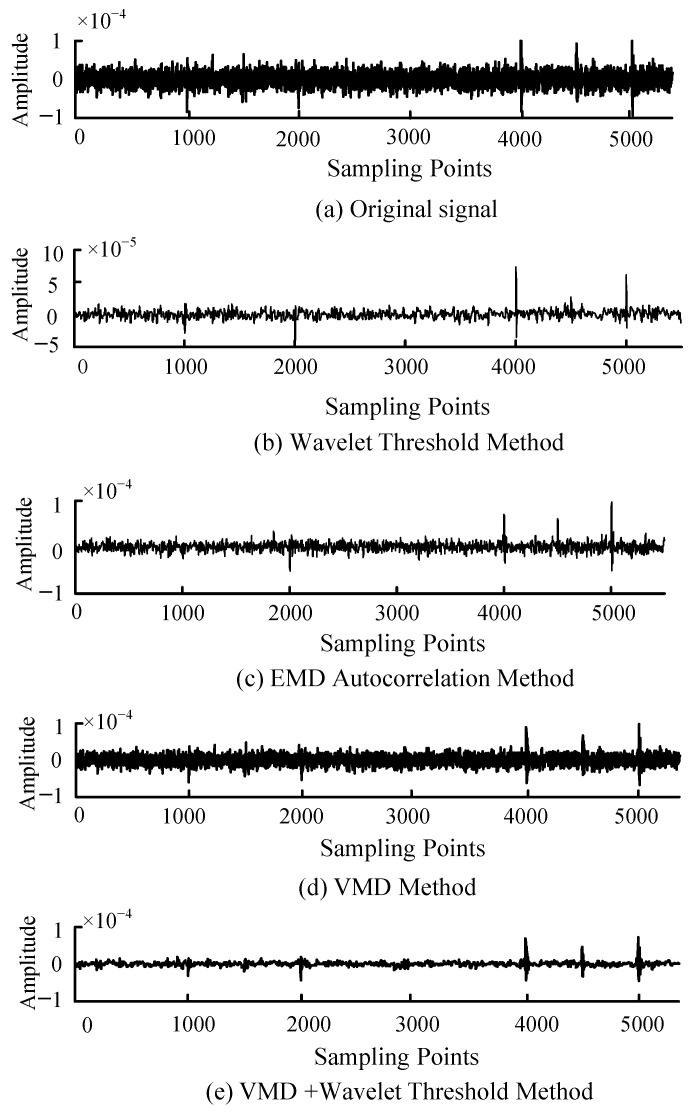
Comparison of four methods of denoising effect (original SNR is −2.673 dB).

The signal *c_k_* processed by wavelet thresholding is reconstructed to obtain the reconstructed signal based on VMD and wavelet threshold. In order to compare the denoising ability of the PD denoising method proposed in this paper, the method is compared with several current mainstream PD denoising methods. Figure 7 shows the reconstructed signal waveform of the noisy PD signal processed by methods such as VMD, wavelet threshold, EMD autocorrelation, and VMD and wavelet threshold. Among them, the EMD autocorrelation method is a denoising method improved by the EMD method, and its denoising effect is better than that of the EMD method. It can be seen that the reconstruction method based on VMD and wavelet threshold is better than the other three methods, and the transient part of the PD signal is well preserved. The denoising effect of the signal processed by the wavelet threshold is also significant, but the transient part of the PD signal is missing more seriously.

Although the denoising ability of various methods can be visualized from Figure 7, in order to make further quantitative comparisons, we denoise the PD signals with different SNR values. The correlation coefficient R and root mean square error RMSE are also introduced as the basis for judging the denoising ability. The detailed calculation formulas of SNR, R and RMSE are shown in Formulas (12)–(14).

(12) $$\mathrm{SNR}=10\log10(\sum_{i=1}^{N}x_{i}{}^{2}/\sum_{i=1}^{N}{\left(x_{i}-x_{i}^{\prime }\right)}^{2})$$

(13) $$R=\frac{\sum_{i=1}^{N}\left(x_{i}-\bar{x_{i}})(x_{i}^{\prime }-\bar{x_{i}^{\prime }}\right)}{\sqrt{\sum_{i=1}^{N}{\left(x_{i}-\bar{x_{i}}\right)}^{2}\cdot \sum_{i=1}^{N}{\left(x_{i}^{\prime }-\bar{x_{i}^{\prime }}\right)}^{2}}}$$

(14) $$\mathrm{RMSE}=\sqrt{\frac{1}{N}\sum_{i=1}^{N}{\left(x_{i}-x_{i}^{\prime }\right)}^{2}}$$

As can be seen from Table 1, the denoising effect of VMD and the wavelet threshold method is significantly better than that of the VMD method, EMD autocorrelation method and wavelet threshold method, especially for signals with smaller SNR values.

## 5. Experimental Signal Analysis

**Figure 8:**
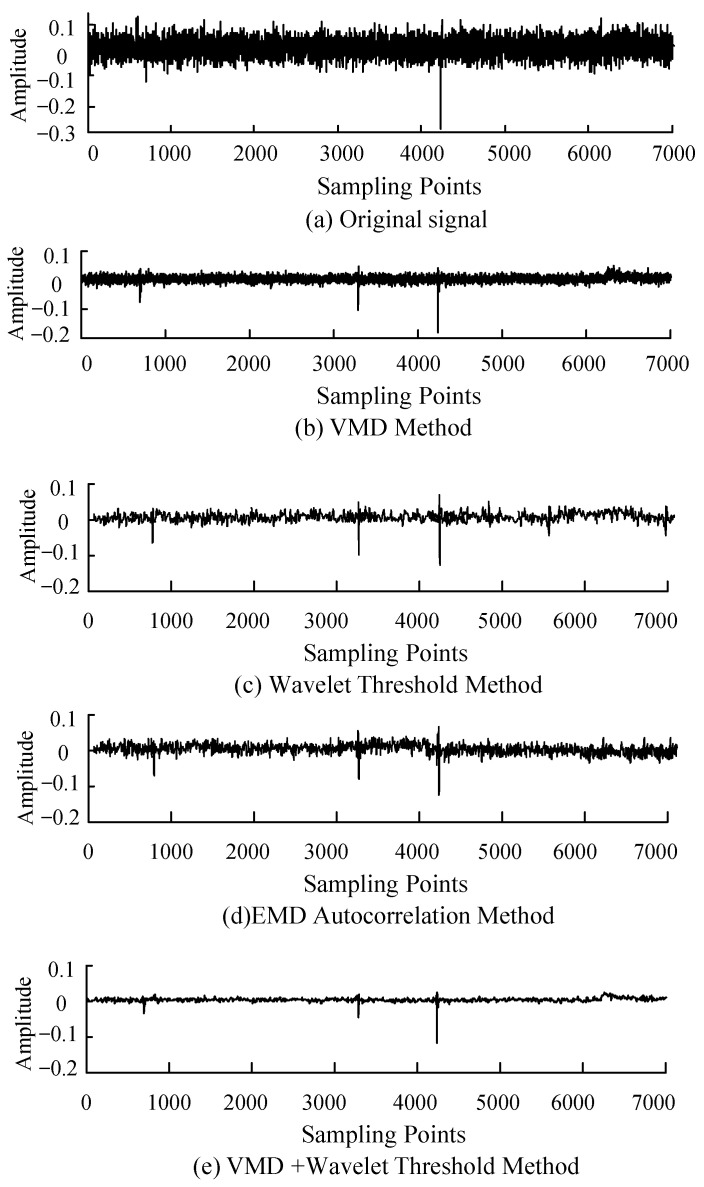
Analysis of PD signal obtained in laboratory.

In order to further verify the denoising effect of the denoising method proposed in this paper on the measured signal, 2.5 kV DC voltage is applied to the 6 kV power cable in the laboratory, the PD voltage signal is measured by the detection impedance method, and the signal data obtained from the experiment are processed by Matlab. Figure 8a shows the waveform of the measured PD signal in the laboratory, and it can be seen that the PD signal is almost drowned in the noise interference. Figure 8b–e are the signals after denoising the experimental signals using the VMD method, wavelet threshold method, EMD autocorrelation method and the method proposed in this paper, respectively.

It can be seen from Figure 8 that the denoising effect of the VMD and wavelet threshold reconstruction method is the best, the PD signal after denoising has no oscillation phenomenon, and the noise is significantly suppressed. Although the wavelet threshold method also plays an obvious role in suppressing the noise, it also causes a large amount of loss in the transient process of the PD signal, which is not conducive to the analysis of the PD signal later. The EMD autocorrelation method and VMD method have limited effects on noise suppression, especially the single VMD method has no obvious effect on noise suppression, and there is still a large number of noise signals. Since the calculation of SNR, R and RMSE requires the original “pure” signal, and the original “pure” signal of the experimental signal is unknown, the Noise Rejection Ratio (NRR) before and after signal denoising is introduced here to measure the denoising effect. NRR characterizes the prominence of the effective signal after denoising. Table 2 shows the NRR calculation results of the two methods.

(15) $$\mathrm{NRR}=10\log10(\sigma_{1}{}^{2}-\sigma_{2}{}^{2})$$

where $\sigma_{1}{}^{2}$ and $\sigma_{2}{}^{2}$ represent the variance of the signal before and after denoising, respectively.

From the calculation results of the NRR of each method in Table 2, it can be seen more intuitively that the NRR of the VMD and wavelet threshold method is the highest, and the NRR of the single VMD method is the lowest. Through the analysis of the NRR of each method, the analysis of the denoising effect of each method in this paper can be supported from another perspective. Combining the results of Figure 8 and Table 2 further proves that the denoising effect of the method proposed in this paper is significantly better than the other three methods.

## 6. Conclusions

The pulse current method is an effective means for monitoring the PD of mining cables. However, when collecting PD signals, due to the influence of on-site working conditions, the noise interference is large, and the cable PD signals cannot be effectively extracted. In order to improve the SNR of the PD signal, this paper proposes a denoising method of mining a cable PD signal based on the optimization of VMD and wavelet threshold. Considering that the number of modal components *K* and the quadratic penalty factor *α* have a great influence on the results of VMD, this paper introduces a genetic algorithm to determine the optimal values of these two parameters, so that VMD can achieve the best results. Meanwhile, according to the respective characteristics of VMD and the wavelet threshold method, these two methods are effectively combined to further improve the denoising effect of a cable local discharge signal. The main conclusions are as follows.

(1) In this paper, by introducing the genetic algorithm, the minimization of the local minimal envelope entropy value is taken as the optimization goal of VMD parameters. Then, the optimal values of VMD parameters are obtained, which avoids the situation that the VMD denoising ability is insufficient due to the artificial setting of the parameter value.
(2) The combined denoising algorithm proposed in this paper combines the advantages of VMD’s ability to adaptively adjust the center frequency of each mode and the excellent time-frequency analysis capability of wavelet threshold. It also avoids the disadvantages of VMD’s weak noise suppression capability and the loss of transient processes by wavelet threshold. Through the experimental comparison, it is found that the method has a more excellent denoising ability, and the filtering performance is better for PD signals with lower SNR.

## Figures and Tables

**Table 1:** Comparison of four ways to suppress the noise effect.

PD Signal SNR	VMD			VMD + Wavelet Threshold Method			EMD Autocorrelation Method			Wavelet Threshold Method		
	SNR	R	RMSE /×10^−6^	SNR	R	RMSE /×10^−6^	SNR	R	RMSE /×10^−6^	SNR	R	RMSE/×10^−6^
−7	−4.29	0.47	9.66	2.79	0.72	4.28	−1.99	0.45	7.41	−0.01	0.50	5.91
−3	0.10	0.64	5.83	5.60	0.86	3.10	1.54	0.62	4.94	2.93	0.71	4.21
0.1	2.43	0.75	4.46	7.01	0.90	2.63	3.70	0.77	3.85	5.15	0.83	3.26
1	3.21	0.78	4.08	7.71	0.92	2.43	4.19	0.79	3.64	5.76	0.86	3.04
4	5.97	0.87	2.97	8.95	0.95	2.11	6.78	0.89	2.70	8.56	0.93	2.20
9	9.33	0.94	2.01	10.6	0.97	1.74	9.78	0.95	1.91	12.06	0.97	1.47

**Table 2:** Comparison of NRR calculation results.

Denoising Method	NRR/dB
VMD method	3.7901
EMD Autocorrelation	4.2705
Wavelet Threshold	4.6294
VMD + Wavelet Threshold	5.3603

## Data Availability

Not applicable.
